# *Campylobacter fetus* infection presenting with bacteremia and cellulitis in a 72-year-old man with an implanted pacemaker: a case report

**DOI:** 10.1186/1752-1947-6-414

**Published:** 2012-11-30

**Authors:** Dragan Ledina, Ivo Ivić, Jakica Karanović, Nenad Karanović, Nikica Kuzmičić, Dubravka Ledina, Željko Puljiz

**Affiliations:** 1 Department of Infectious Diseases, University Hospital Split, Split, Croatia; 2 Department of Microbiology and Parasitology, University Hospital Split, Split, Croatia; 3 Department of Anesthesia and Intensive Care, University Hospital Split, Split, Croatia; 4 General Hospital Pakrac, Pakrac, Croatia; 5 Department of Oncology, University Hospital Split, Split, Croatia; 6 Department of Gastroenterology, University Hospital Split, Split, Croatia

## Abstract

**Introduction:**

*Campylobacter* is an important causative agent of intestinal infections in humans. Bacteremia is detected in less than 1% of patients, mainly in immunocompromised patients and in extreme age groups. Cellulitis is a relatively common manifestation of *Campylobacter* infection, but concomitant bacteremia is a rare event. Infections of the pacemaker area are caused primarily by staphylococci, followed by fungi, streptococci and Gram-negative rods. To the best of our knowledge, this is the first case report of pacemaker pocket infection and bacteremia caused by *Campylobacter fetus*.

**Case presentation:**

A 72-year-old Croatian Caucasian man with myelodysplasia, impaired fasting glucose levels and a recently implanted permanent pacemaker was admitted to hospital after six days of fever, development of red swelling of the pacemaker pocket area and worsening of his general condition. No antibiotic therapy was introduced in the outpatient setting. He denied any recent gastrointestinal disturbances. With the exception of an elevated leukocyte count, erythrocyte sedimentation rate, and C-reactive protein and blood glucose levels, other laboratory findings were normal. Treatment with vancomycin plus netilmicin was introduced, and a surgical incision with drainage of the pacemaker pocket was performed. The entire pacemaker system was removed and a new one re-implanted after 14 days of antibiotic therapy. Transesophageal echocardiography showed no pathological findings. Three subsequent blood cultures obtained on admission as well as swab culture of the incised pacemaker area revealed *Campylobacter fetus*; stool and pacemaker lead cultures were negative. According to the microbiological results, antibiotic therapy was changed to ciprofloxacin plus netilmicin. A clinical examination and the results of a laboratory analysis performed after two weeks of therapy were within normal limits.

**Conclusion:**

Myelodysplasia, impaired fasting glucose levels and older age could be contributing factors for the development of bacteremic *Campylobacter fetus* cellulitis. Emergent surgical and antibiotic treatment are mandatory and provide the optimal outcome for such types of pacemaker pocket infection.

## Introduction

To date, 15 species of *Campylobacter* are known. *Campylobacter* is an important cause of intestinal infection in humans. These microorganisms sporadically cause bacteremia or extraintestinal infections [1]. Less than 1% of patients with diarrhea caused by *Campylobacter* species develop bacteremia. Invasive disease usually occurs in immunocompromised patients and in persons at the extremes of age [2]. Contaminated poultry, meat and unpasteurized milk are usual sources of *Campylobacter* infection, but direct spreading from infected animals is also possible. Intestinal infections are most commonly caused by *C. jejuni*[3]. Extraintestinal manifestations are rare and include meningitis, endocarditis, septic arthritis and osteomyelitis [4,5]. *C*. *fetus* has been recognized as the major causative agent of extraintestinal infections. Cellulitis is a relatively common manifestation of *Campylobacter* infection but concomitant bacteremia is rare [6]. Infections of the pacemaker area are primarily caused by staphylococci, followed by fungi, streptococci and Gram-negative rods [7]. Retention of an infected pacemaker system is associated with high mortality [7].

Cases of *C*. *fetus* endocarditis [1], as well as infection of an internal cardioverter defibrillator [8], have previously been described. To the best of our knowledge, only one article regarding pacemaker site *Campylobacter* infection has been published thus far [9]. We report the case of a patient with myelodysplasia and diabetes mellitus who, 14 days after implantation of a permanent pacemaker, developed bacteremic cellulitis of a pacemaker site infection caused by *C*. *fetus*.

## Case presentation

A 72-year-old Croatian Caucasian man was admitted to our hospital with fever of up to 40°C, inflammation of the pacemaker pocket area and progressive general weakness. Five years prior to this hospital admission, our patient was diagnosed with myelodysplastic syndrome and he had an impaired fasting glucose level that was treated with diet only. The current symptoms commenced two weeks after the implantation of a permanent pacemaker. He developed low grade fever up to 37.5°C accompanied by general weakness. On the seventh day of his disease, fever with chills rose up to 40°C and he was referred to our Department of Infectious Diseases. He denied any gastrointestinal disturbances including diarrhea or abdominal pain during or before this illness. He also denied close contact with animals and eating of undercooked meat or unpasteurized milk. He received no antibiotic therapy in the outpatient setting.

On physical examination there was painful red swelling of the pacemaker area, 15cm in diameter, with dehiscence of the surgical suture and serous leakage evident on his right chest region. His heart rhythm was regular with a systolic heart murmur, but our patient was hemodynamically stable. His arterial pressure was 140/70mmHg, and heart rate 84 beats/min. Bronchial rales were heard on the right side of his chest. Hepatomegaly and splenomegaly were not registered. Laboratory tests revealed an erythrocyte sedimentation rate of 96mm/h. His leukocyte count was 17.9×10^9^/L (86% polymorphonuclear cells), erythrocytes 2.86×10^12^/L, hemoglobin concentration 9.9g/dL and platelet count 242×10^9^/L. His C-reactive protein level was 156μmol/L, and blood glucose level 9.06mmol/L. Other blood biochemical parameters (creatinine, blood urea nitrogen, total protein, albumin, globulin, immunoglobulin, amylase, serum aspartate aminotransferase, alanine aminotransferase, bilirubin, creatine phosphokinase and alkaline phosphates) were within the reference ranges. A chest X-ray showed no pulmonary infiltrations. Transthoracic, as well as transesophageal echocardiography showed no signs of endocarditis. Blood, urine and wound swabs were obtained and vancomycin plus netilmicin introduced as empiric therapy. Swab culture of the infected region and three consecutive blood cultures revealed *C. fetus*. Urine, stool and pacemaker lead cultures obtained later, were negative.

Cultures of swabs and blood samples, plated on charcoal-based blood-free selective medium, incubated under microaerobic (5% O_2_, 10% CO_2_ and 85% N_2_) conditions at 25°C and 35°C resulted in the growth of gray, flat, irregular, spreading colonies after 48 hours.

The Gram stain showed a characteristic appearance of Gram-negative, curved to S-shaped rods. The identification of the strain was performed on the basis of conventional biochemical tests. An antibiotic susceptibility test, performed by using the E-test (PDM Epsilometer; AB Biodisc, Solna, Sweden), gave these minimum inhibitory concentrations for the following antimicrobial agents: ampicillin 0.12mg/L, tetracycline 0.25mg/L, erythromycin 0.19mg/L and ciprofloxacin 0.064mg/L. Minimum inhibitory concentration breakpoints were interpreted according to the guideline established by the British Society for Antimicrobial Chemotherapy.

Our patient was transferred to our Department of Cardiology where a local surgical incision and removal of the permanent pacemaker by simple traction were performed. The pacemaker pocket was drained and the wound closed by secondary sutures. Surgical drainage was followed by an immediate drop in the fever. According to the antibiotic susceptibility of *C*. *fetus*, therapy was switched to ciprofloxacin plus netilmicin. Our patient’s condition improved quickly following the treatment. Repeated blood cultures obtained during and at the end of the second week of therapy were negative. A new pacemaker system was re-implanted 14 days after his admission to our hospital, at the contralateral side of his thorax. Three days later, our patient was discharged from hospital. One month later, the area of cellulitis had healed completely, and there were no signs of inflammation of the contralaterally implanted pacemaker.

## Discussion

This is the first case report of pacemaker pocket infection and bacteremia caused by *C*. *fetus*. Bacteremia caused by *C*. *fetus* is a rare event, particularly in immunocompetent hosts [5]. *C*. *fetus* has an affinity for vascular endothelium, and different cardiovascular complications such as endocarditis, thrombophlebitis, pericarditis and mycotic aneurysms have been reported [1,5]. Some degree of immunodeficiency appears to be mandatory for the development of extraintestinal *Campylobacter* infections. *C*. *fetus* bacteremia is a serious infection that is often accompanied by relapses and complications despite the prompt administration of the appropriate antibiotics [5]. Human immunodeficiency virus infection, alcoholism, cirrhosis, old age, pregnancy, diabetes mellitus, malignancies, beta-thalassemia, systemic lupus erythematosus, inherited immunodeficiencies, transplantation and splenectomy have all been recognized as important risk factors for the development of serious *C*. *fetus* infection [5,6]. In our patient, diabetes mellitus and myelodysplasia could have been contributing factors to the severe pacemaker pocket infection and bacteremia. Another recognized cause of serious extraintestinal infections is *C*. *lari*. After reviewing the literature, we found one case of pacemaker infection caused by *C*. *lari*[9]. In eight cases of *C*. *lari* bacteremia, only two had gastrointestinal symptoms [9,10]. Isolation of *C*. *lari* and *C*. *fetus* in several cases of prosthetic device infections encouraged an opinion of their propensity to cause such types of infections [11,12].

Although in our case, an intestinal source of infection was not proven, it cannot be ruled out as a stool culture was obtained during empiric therapy that included an aminoglycoside. The negative culture of the pacemaker lead could also be a consequence of previously introduced aminoglycoside therapy. Therefore, the mode of pacemaker pocket infection for our patient remains unclear: direct inoculation during insertion of the pacemaker or hematogenous spread originating from an asymptomatic intestinal infection.

Most authors recommend a combination of removal of cardiac devices, either surgically or by a transvenous route, with antimicrobial therapy [13]. Percutaneous removal of infected pacemakers combined with appropriate antibiotic therapy is safe and effective, as shown in our case. Both simple traction and countertraction techniques can be used with low risk of complications [14]. The mortality of patients with retained infected pacemaker systems is high. Therefore, removal of both the pacemaker generator and leads is essential in case of an infection. Fortunately, as in our case, pulse generator pocket infection is quite rarely complicated by infective endocarditis [15].

## Conclusion

*C*. *fetus* is a rare but possible cause of serious pacemaker pocket site infection. In particular it should be considered as a causative agent in patients with underling illnesses that are contributing factors for a wider spectrum of causative agents. The best treatment results are obtained with a combination of surgical and antimicrobial therapy.

## Consent

Written informed consent was obtained from the patient for publication of this case report and accompanying images. A copy of the written consent is available for review by the Editor-in-Chief of this journal.

## Competing interests

The authors declare that they have no competing interests.

## Authors’ contributions

DrL, II and NeK were involved in the primary care of the patient. DrL and DuL analyzed and interpreted the patient’s data. JK performed the microbiological analysis. DrL was a major contributor to the draft manuscript, which was later edited by NiK and ŽP. All authors read and approved the final manuscript.
